# Psychology of Descartes
*Discourse on the Method of Rightly Understanding the Reason: and Meditations on the First Philosophy. From the French of Descartes. Simpkin and Marshall, 1851-3.


**Published:** 1855-01-01

**Authors:** 


### Art. IV.—PSYCHOLOGY OF DESCARTES.*

Descartes stands at the head of the whole continental school of speculative philosophy, as Bacon is the presiding genius of all our own science. To speak of the schools of Locke and Reid (which have been mainly inspired by the Baconian spirit) as *sensational*, we regard as an injustice. The real view of the case is, that among ourselves the inductive mode of pursuing knowledge has mainly prevailed over the theoretic. Even Dugald Stewart, after Reid had established the intuitive and *à priori* character of the ultimate grounds of truth, still speaks of the "Inductive Philosophy of the Human Mind;" and very properly: for we only know that any first principle is an element of the psychology of man, by finding that it is not peculiar to our own minds or to a few others, but is common to the race: in other words, we know the principle as a psychological generalization, by induction. Now, on the continent, so far as the Cartesian tendencies have been followed up, the aim, in psychological speculation, has been generally to seize upon some one or more principles supposed to be intuitive and ultimate, and thence to deduce a whole system of metaphysics—such as it has sometimes been—independently of experience.

In like manner, however, as we dislike the epithet "sensational," as applied to characterize the psychology of the Lockian and Reidean schools, just because it plainly fails to describe them—so we should object to attempt drawing too sharp a line of demarcation between these schools and that of Descartes, especially as represented by himself and some of his less adventurous followers. That many who acknowledge him as their first leader have gone into an unrestrained idealism, everybody knows; but we shall be safe if we say that while the Baconian school of philosophy, whatever subjects it may happen to cultivate, is marked by a closely inductive spirit, which admits first principles, indeed, (as all schools must,) but admits them cautiously; the method professedly derived from Descartes has often really exhibited (according to his own example) a signal deviation from its master's best rules, and has, by a misapplication of the deductive process, often landed philosophers, however "pitiless" their logic, in absurdities which were certainly pitiful enough, and which soon ceased to have any authority when once their fashion was over.

Descartes would no doubt have been as much surprised to find himself charged with being the prime agent in leading the way to the German Pantheism, as Locke would in having laid at his door the

* Discourse on the Method of Rightly Understanding the Reason: and Meditations on the First Philosophy. From the French of Descartes. Simpkin and Marshall, 1851-3.

gross materialism of Cabanis, and all the horrors of the old French revolution. It is easy enough for ingenuity, inspired with the determination of finding everywhere a system, to refer the most extreme opinions back to sources which have really little to do with them, and which are truly due only to perversions and distortions for which the original sources are not fairly responsible. We say this, because not only Locke, but Descartes also, has, in our judgment, been rather too hastily identified with speculators of a date remote from his own age; though we have never doubted that, notwithstanding his exalted merit, he has, in all conscience, a sufficient amount of error to answer for on his own account.

Descartes was born of a good family at La Haye, in Touraine, in 1596; and was educated by the Jesuits in the neighbouring seminary of La Flèche. According to the fashion of his rank in life, he entered the army, and is said to have fought very bravely at the battle of Prague, in 1620. He afterwards travelled in Holland, France, Italy, and Switzerland. On his return, he sold a part of his patrimony in France, and retired to Holland, in order to devote himself, in seclusion, to the philosophical and mathematical inquiries to which he was addicted. As the fame of his discoveries and speculations increased, he became obnoxious to the Church, though no one could be more obsequious to it, and he was exposed to some danger in consequence; so that he was glad to accept an invitation to reside at Stockholm, from Christina, that very eccentric young queen of Sweden, whose passion it was to surround herself with foreigners, chiefly Frenchmen, of whom some were true *savans* and others mere pretenders. Assured of a safe asylum from his enemies, in Sweden, Descartes repaired thither in 1649; but the coldness of the climate was more than the delicacy of his constitution could bear, and he caught a cold in one of the unseasonably early morning visits which his royal pupil exacted of him—the hour of five being fixed on for the lesson—and he died of peripneumony in 1650. His remains were taken to France, and interred with great ceremony in St. Geneviève du Mont.

Perhaps there are few more striking examples of the strength and weakness of human reason than Descartes. He was an apt student at his college of the learning of the day, and took large draughts of the *encyclicæ disciplinæ*, as set forth in the *trivium* and the *quadrivium*, according to the fashion of the age.* Yet such was the precocious independence of his mind, that, on quitting La Flèche, he declared that all the benefit he had derived from his college course was a profound conviction of his own ignorance, and as profound a contempt for the reigning philosophy of the schools. Hence he resolved to enter on a system of independent research, and to doubt of everything until he had in some way convinced himself of its truth. But though he thus set out with what may be called, in the best sense, a universal scepticism, many of his theories are based on the most unphilosophical credulity; so that it has not been said without reason that he "began by doubting everything, and ended by believing anything."

Descartes published his *Discours de la Méthode pour bien conduire sa Raison, et chercher la Vérité dans les Sciences*, in 1637; his *Meditationes de Prima Philosophia*, in 1641; and in the same year came out his *Responsiones ad Objectiones*. The *Principia Philosophiæ* was published in 1647; and the work entitled *Les Passions de l'Ame*, in 1649, not long before his death. These writings contain the principles of his psychological and metaphysical philosophy, as also of his natural philosophy and cosmology; and his treatises, *La Dioptrique*, *les Météores*, and *La Géométrie*, were given as illustrations of his method. Posthumously were published, *Regulæ ad Directionem Ingenii*, and *Inquisitio Veritatis per Lumen Naturale*.

The preliminary to Descartes' method is *doubt*, or neutrality as to belief or disbelief,—in fact, suspension of judgment; not, indeed, the captious uncertainty of the sceptic, which makes doubt an end in itself, identifying it with the absence of all truth and effective conviction: the Cartesian doubt, on the contrary, is to prevent error, and is not merely negative; it is to lead to truth. This scientific doubt is of course opposed to the dominion of all mere authority in the sphere of the true and the false. Not only, therefore, must great names here go for nothing, but all the convictions which have grown up from infancy, by education and habit, are to be held in abeyance. How fatal all this must have been to the lingering scholasticism of the early part of the seventeenth century, is evident! But the hour of scholasticism had already arrived, and Bacon and Descartes had each a mighty hand in prostrating it for ever. What the former did in England, the latter did on the continent of Europe. There is evidence that Descartes was acquainted with the writings of Bacon before he had published anything of his own; but the independence of his mind and the originality of his method are not affected by this fact. Descartes tells us that he had begun to seek truth as far as possible for himself, independently of all authority, as early as 1619; which was rather sooner than the first publication of Bacon's great work, the *Novum Organon*. Descartes' method, too, in its application and development, leaned in an opposite direction to that of Bacon, wherever a choice of procedure was possible in the pursuit of scientific inquiry. From this source we trace not a few of the egregious errors in physical science which the progress of knowledge has utterly exploded, but which Descartes propounded with a full conviction of their truth. Bacon's method was decidedly inductive and practical, Descartes' was deductive and theoretical. Bacon sought for causes from their effects; Descartes says in so many words, "I seek not causes from their effects, but effects from their causes."

Much as there is to except against the method of Descartes in many of the cases in which he has applied it, nothing can be more beautiful than the child-like openness and simplicity of mind with which he describes his first attempts, at nineteen years of age, to revise all his opinions, and, if possible, to detect those which were erroneous, and set those which were true on a sound basis. It is quite refreshing to find so great a mind so completely divested of all dogmatism and so open to conviction, so willing to unlearn as well as to learn. Is not this, however, what we might expect from an intellect of the highest order? There are men who see no difficulties; why so? Because they do not see far enough and deep enough. They have not enough comprehension to be good doubters, and they have not humility enough to allow themselves to be corrected and convinced of error. Not so Descartes; and if his subsequent career might seem little in keeping with his earlier caution and cool judgment, it was that he became so passionately enamoured of his method, that his imagination at last fairly ran away with his discretion, more especially as regarded his speculations in physical science.

"From my childhood I have been familiar with letters, and as I was given to believe that by their help a clear and certain knowledge of all that is useful in life might be acquired, I was ardently desirous of instruction. But as soon as I had finished the entire course of study at the close of which it is customary to be admitted into the order of the learned, I completely changed my opinion, for I found myself involved in so many doubts and errors, that I was convinced I had advanced no further in all my attempts at learning, than the discovery, at every turn, of my own ignorance. I revered our theology, and aspired as much as any one to reach heaven; but being given assuredly to understand that the way is not less open to the most ignorant than to the most learned, and that the revealed truths which lead to heaven are above our comprehension, I did not presume to subject them to the impotency of my reason, and I thought that in order competently to undertake their examination, there was need of more special help from heaven, and of being more than man. Of philosophy I will say nothing, except that when I saw that there is not a single matter within its sphere which is not still in dispute, and nothing, therefore, which is above doubt, I did not presume to anticipate that my success would be greater in it than that of others; and, further, when I considered the number of conflicting opinions touching a single matter that may be upheld by learned men, while there can be but one true, I reckoned as well-nigh false all that was only probable. And as to the other sciences, inasmuch as these borrow their principles from philosophy, I judged that no solid superstructures could be reared on foundations so infirm; and neither the honor nor the gain held out by them was sufficient to determine me to their cultivation, for I was not, thank heaven, in a condition which compelled me to make merchandize of science for the bettering of my fortune; and though I might not profess to scorn glory as a cynic, I yet made very slight account of that honor which I hoped to acquire only through fictitious titles. And, in fine, of false sciences I thought I knew the worst sufficiently to escape being deceived by the professions of an alchymist, the predictions of an astrologer, the impostures of a magician, or by the artifice and boasting of any of those who profess to know things of which they are ignorant." — *Discours de la Méthode*, part the first.

The upshot of this state of mind in our young philosopher was, that he resolved to shut up his books, and to study the great volume of the world and *himself*. He spent some years in travelling, and in courts and armies, and in trying to learn something from everything. He then, as he tells us, resolved to turn his attention home upon his own inward consciousness; to endeavour to reject all the opinions and modes of thinking which seemed rather due to fashion and education than to reason, and to construct for himself a new edifice of knowledge out of those materials, only, which he had tried and tested to the utmost of his power: for a comparison of social life and current ideas, in the different nations and grades of society with which his travels made him acquainted, had led him to "infer that the ground of our opinions is far more custom and example than any certain knowledge," and to "remark, that a plurality of suffrages is no guarantee of truth where it is at all of difficult discovery." Descartes thought, further, that if he could only fulfil his unwavering determination to bind himself down to the four following laws, in the conduct of his understanding, he should find them quite sufficient as a guide to the discovery of truth. His rules were: "*first*, never to accept anything as true which I did not clearly know to be such; *secondly*, to divide each of the difficulties under examination into as many parts as possible; *thirdly*, to conduct my thoughts in such order as to commence with the simplest and easiest objects, and so to ascend by degrees to the knowledge of the more complex; *fourthly*, in every case to make enumerations so complete and reviews so general, that I might be assured nothing was omitted." True enough these rules are good; but the difficulty is to make sure of reducing them to practice, especially the first and the last.

Descartes was, probably, the first philosopher who laid down the position, in formal terms, that to every person of the least reflection there is one truth more unassailable than any other—namely, his own personal existence. No matter what may be our ontological theory of body or of mind, our conviction that we *are* remains always the same. A man may say that mind is only a function of matter, or that mind and matter are identical, or that body is nothing but force or centres of force, or that the whole material universe is an illusive ideal panorama and not a reality; he may be a disciple of Berkeley, or of Leibnitz, of Fichte, Schelling, or Hegel, or of Cabanis and the materialists; he may begin with one of these opinions, and successively adopt each, and go the round of them; but amidst all the transmigrations through which his opinions may pass, all the metamorphoses of his psychological system—amidst all scepticism, all dogmatism, all pantheism,—in short, all the phases of his belief and his philosophy, he never can for a moment doubt that all these changes are changes of *himself*, that there is a *me* which undergoes them, and that this *me* is conscious of itself. This was the truth of which Descartes pronounced that it is intuitively, irresistibly, and irrevocably certain, admitting of no doubt; since, if the absurdity could be imagined of a man doubting his own existence, the very doubt itself is an act which involves the conscious existence of the doubter. As a general rule, indeed, all philosophical inquiry must, wherever possible, according to Descartes, be preceded by doubt. Once in our lives, he remarks in his *Principia*, we should doubt of everything as far as we can, in order to discover truth—doubt of whatever admits the possibility of the question, "is it true, or is it not?" He found that he could doubt of everything which his senses appeared to teach him. "All this might possibly be a delusion; for the senses do frequently lead us astray—witness optical illusions." Again, the conclusions of the understanding, however certain they might eventually turn out to be, at all events admitted of inquiry as to their validity before they should be received as certain truth. "Thus, rejecting all those things concerning which we can in any way doubt, and imagining them to be false, we may assume that there is no God, no sky, no bodies—that we ourselves have neither hands nor feet, nor in fact a body; yet we who devise to ourselves such cogitations cannot imagine that *we are nothing*, for it is a contradiction that we should think and not exist." Hence the truth, *ego cogito ergo sum*, the first and most certain truth which presents itself to any one who seriously sets himself to philosophical thinking. "Hence," says our author, in the fourth part of the *Discours de la Méthode*, "though I resolved that all things which had entered my mind were not more true than my dreams, yet it was necessary that I, who thought, must be something. This truth is so firmly assured that it can never be shaken by sceptics; and I judged that I might receive it as a first principle of the philosophy I sought. I could suppose that I had no body, that there was no world, no place where I was, but not that *I* was not."

It is proper here to remark that Descartes did not lay down his first principle, *Je pense, donc je suis*, as a logical argument, an enthymeme (according to the more modern use of this term) or syllogism with one of the premises (here the major) suppressed. This would have clearly involved the *petitio principii* which Gassendi and others hastily charged him with in the use of this aphorism. Spinoza, the learned and accurate commentator on Descartes, has justly remarked, in his work entitled *Renati Descartes Principia Philosophiæ, more Geometrico Demonstrata*, that he only meant to state the fact that our thinking is attended with an irresistible conviction of our existence. For Descartes himself, in his *Responsio ad Secundas Objectiones*, says in so many words,—"I think, therefore I am, or I exist, is not concluded by force of a syllogism, but as a thing self-evident."

Having convinced himself that this one truth might be regarded as utterly beyond all question, Descartes proceeds, in his Discourse on Method, to inquire why he could not but admit it, and in general what is required for a proposition to be regarded as true? He replies,—"I found that, in '*I think, therefore I am*,' there was nothing to induce me to believe it true excepting that I see clearly that, *to think, it is necessary to be.* I then concluded that all the things which we conceive very clearly and distinctly are true, and that there is only some difficulty in well-remarking what those things are which we conceive distinctly." Hence, it is evident that our illustrious philosopher makes consciousness the point of departure for the discovery of all other truth. He therefore laid down as a fundamental element of his system, that *all our ideas which are perfectly distinct are true.* In his *Principia*, he calls this axiom the "foundation of all science, and the measure and rule of truth." It was even the secret basis of his conviction of his own existence. He believed this latter truth, because "whatever is clearly and distinctly conceived of as existing, and cannot but be so conceived when thought of, must really exist." How wide a field this axiom may open to the illusions of imagination, prejudice, and self-will, must be evident to the reader! Leibnitz subsequently tried, with whatever success, to limit and rectify this somewhat ominous element in the Cartesian philosophy.

Our author further tells us that those chains of geometrical reasoning by which the most difficult demonstrations are reached, led him to the conclusion that a similar procedure should be applied to all human knowledge; and that if we only take care not to admit anything as true which is not so, and preserve the proper order of deduction, like the mathematicians, we may attain to all the truth which men can know.

It is evident from the above that four separate elements meet us on the threshold of the Cartesian psychology; namely, that all our knowledge ought to be preceded, as far as possible, by previous doubt; that there is one fact which we cannot doubt of, which is, our own existence, the primary and most indubitable of all truths to every thinking being—for himself; that the criterion of this and all other real truths is the perfect clearness and distinctness with which it is apprehended; and that the method which we should always try to employ in the pursuit of science and philosophy, is the mathematical or deductive. In reference to this last principle, Descartes says, in the third part of his *Principia*, that his object is to "*deduce effects from causes, and not causes from effects.*" This remark shows how much he leaned to the *à priori* method of inquiry, and how different a tendency his philosophy exhibits in the outset from that of Bacon, who made *induction*, or the *à posteriori* method, everything. We say "tendency;" for neither could Descartes confine himself wholly, in the development of his principles, to pure deduction, nor Bacon, on the other hand, fail of applying intuitive, or *à priori* elements in dealing with his inductions. In the doctrine of causation the two principles may be said to meet. In concluding by induction we establish a general fact by bringing in a certain number of instances; and we then assert, that wherever the like instances occur again they are to be traced to similar causes. Hence, even in induction we assume the uniformity of the laws of nature; which is only another way of saying that like causes, in like circumstances, produce like effects.

Descartes, in illustration of his method, applies it to the proof of the existence of a Deity. The arguments on this subject are stated the most clearly and with the greatest condensation in Descartes' *Responsio ad Secundas Objectiones.* We will give them nearly in his own words:—"First, the existence of God is known from the consideration of his nature alone. *Demonstration:* To say that an attribute is contained in the nature or concept of a thing, is the same as to say that this attribute is true of this thing, and that it may be affirmed to be in it; but necessary existence is contained in the nature or concept of God; hence we may say with truth, that necessary existence is with God, or that God exists. Secondly, the existence of God is demonstrated from this alone, that his idea is in us. *Demon-*

*stration*: The objective reality of each of our ideas requires a cause in which this same reality is contained, not simply objectively, but formally and eminently: but we have in us the idea of God, and the objective reality of this idea is not contained in us, nor can it be contained in any other except in God himself. Thirdly, the existence of God is also demonstrated from this, that we ourselves, who possess the idea of him, exist." Here Descartes employs a somewhat tedious *sorites* (which we omit for the sake of brevity) with a view to prove the conclusion, from our "not having the power of self-conservation, and so being conserved by another who has in himself all the perfections that are wanting in us, and thus being God."

The first of these arguments is *ontological*, or founded on the very nature or essence of the idea we have of God, which is "that of a Being omniscient, all-powerful, and absolutely perfect. In this idea there is contained existence absolutely necessary and eternal. The equality of its three angles to two right angles is necessarily comprised in the idea of a triangle, and the mind is firmly persuaded of this equality; so, from its perceiving necessary and eternal existence to be comprised in its idea of an all-perfect Being, it ought to conclude that he exists."* The second argument is the *psychological* one. It is founded simply on the fact that we have, or are capable of having, an idea of an all-perfect Being in our minds, and with the greatest possible clearness and distinctness, however inadequate this idea may be.† Now it is tolerably evident that both these arguments, though they have been distinguished by name, are, in strictness, the same: they are both psychological; they are based on our *conceptions*. We have certain ideas of a Supreme Being, "therefore he exists." As to the third argument, it has confessedly, at the base of it, the doctrine of causation. "From whom could I," asks Descartes, "derive my existence, if there were no God?"‡ He decides that he could not, on that supposition, have been preserved in being, nor, indeed, have existed at all. The last argument is less Cartesian than the rest in its basis, though it also is blended with trains of *à priori* or deductive reasoning.

We have long been convinced that every genuine argument in proof of a Deity must ultimately resolve itself into some form of the doctrine of causation; and we think so still, after again reviving our converse with Descartes. We apprehend that Descartes' argument from the *clearness* of our idea of a Deity, and from what is the necessary analysis of that idea, is assailable on many sides. The utmost that we can say in this direction, if we wish to base our inquiries on

* Princip. Philos. XIV. Meditat. V. † Meditat. III. ‡ Ibid.

a psychological principle common to mankind, is, that all men have a notion of power beyond human: but this notion may be polytheistic, fetish, degraded in the extreme by its adjuncts—as well as monotheistic and Christian. Grant even that a very clear idea of one infinite Supreme were universal, may we not ask, "does a clear conception of a thing guarantee its existence—a mountain of glass, for instance? Descartes anticipated this objection, and he offers a reply to it, in his Fifth Meditation, as follows:—"I cannot conceive God unless as existing; it follows that existence is inseparable from him: not that this is brought about by my thought, but, on the contrary, the necessity which lies in the thing itself determines me to think this way: for it is not in my power to conceive a God without existence." Thus Descartes makes the ontological argument corroborate the psychological; but is the elucidation satisfactory? We think not. Descartes further explains:—"It is not in my power to conceive a God without existence, that is, a Being supremely perfect, and yet devoid of absolute perfection: as soon as I discover that existence is a perfection, I infer the existence of this first and sovereign Being. I can conceive no other being except God, to whose essence existence belongs." Descartes, in short, maintains that the idea of God is psychologically innate, *née avec moi*, in the highest sense in which the term innate can reasonably be used; and that, ontologically, necessary existence is essential to the very idea of God. Now we would venture to say that what is truly "innate" (in the Cartesian sense)—we would rather say *intuitive*—is the principle of *causation*: to believe that every change must have a cause is constitutional to the human mind, and this principle lies at the basis of rational religion. The ontological argument of Descartes, so far as distinct from the psychological, is a *petitio principii* so evident, that nothing but the passion which he had for *à priori* reasoning, or the deductive method, as though it were almost everywhere applicable, could have prevented him from seeing it. We repeat our conviction that the principle of causation will be found lying, in some form or other, at the basis of all satisfactory evidences of the Divine existence. We are capable, no doubt, of forming some sort of conception of a Being infinite, eternal, all-perfect. Whence this range of thought, only the grander and the more sublime because we can sufficiently measure it with its object to know how limited it is? Whence those faculties of man? Whence came they, what is their origin, their cause? But our space will not allow us to pursue this topic.

Descartes further held the existence of God to be the basis of all other truths. Even geometrical demonstrations have no other foundadation than his existence.* "If I did not know that there is a God, I might readily come to doubt of the truth demonstrated that the three angles of the rectilineal triangle are equal to two right angles. But after I have discovered that God exists, as I at the same time observed that all things depend on him, and that he is no deceiver, I thence infer that all which I clearly and distinctly perceive is of necessity true; and on the right conception of the existence of a Supreme Being the certitude of all other truths is so absolutely dependent, that, without this knowledge, it is impossible ever to know anything perfectly."† This theory of truth, we must venture to say, is, at the least, exceptionally expressed. Not that there is any doubt that, as God is the author of all created being, the relations of things, not excluding those of mental phenomena, are so far dependent on him. Nether is it "possible for God to lie." Yet man may deceive himself, or be deceived, sometimes even when he thinks his mental vision the clearest. Again, is there not a *nature of things* which we cannot suppose altered, under any circumstances? Can we imagine it possible, in any time, or in any world, that a triangle can be conceived which should not retain its existing properties? If we receive some of the statements of Descartes as they stand, a speculative atheist must, as such, ever remain ignorant of geometry!

Descartes' theory of our knowledge of a Deity, and of innate ideas in general, has been sometimes much exaggerated; though, it must be confessed, his phrase *née avec moi* was not well-chosen to express what he really meant. Voltaire, in his thirteenth Letter "On the English Nation," says that our author asserted that "the soul at its coming into the body is informed with the whole series of metaphysical notions, knowing God, infinite space, possessing all abstract ideas." Not so. Descartes denies, altogether, that he meant any such thing. In his "Reply to the Objections of Hobbes," he explains *ideæ innatæ* as those ideas which the mind has the faculty of eliciting for itself.‡ In the ninety-ninth Letter of the first part of his own Correspondence, he uses even still more qualified and popular language, stating that when he said the idea of God was innate in us, he never meant more than that nature had endowed us with a faculty by which we may know God. "I never said, or thought," he adds, "that such [innate] ideas had an actual existence, or even that they were *species* distinct from the faculty of thinking." The latter clause of the remark is quite in harmony with Descartes' general repudiation of the ancient

* And, we may add, his *will;* for, according to Descartes, the equality of the three angles of a triangle to two right angles, is a consequence of the will of God; hence the proposition is true and cannot be otherwise.

† Meditat. V.

‡ Denique cum dicimus ideam aliquam esse innatam, intelligimus tantum nos habere in nobis facultatem illam eliciendi.

ideal theory, that of images or species existing in the sensorium. He defines ideas as being "all that is in our mind when we conceive a thing, in whatever way;" and he distinguishes them into three sorts, (*adventitious*, as the common idea of the sun; *factitious*, as the idea of the sun which astronomy gives us; and *innate*, as the idea of God, of mind, of a triangle,) as may be seen in his Meditations. In his *Traité des Passions*, he classifies ideas (by a phraseology which appears to us not very happy) into forms of thought, of passion, and of will.

In our philosopher's theory of *substance* we see a germ of Spinozism. A substance he held to be that which exists *really*, the Deity alone being such in a proper sense—a true substance requiring nothing besides itself for its existence, while all else can exist only by its concurrent energy.* There are two kinds of finite, created or secondary substances—matter, and mind or soul. The nature of matter consists solely in its being something extended, the extension of which does not differ from the thing itself which is extended.† "Matter and extension are the same thing." ‡ Here it would seem that matter and extension are completely identified by Descartes. He says that extension in three dimensions constitutes the *nature* of bodily substance. "Extension alone remains," he says, "when we reject hardness, colour, weight, heat, cold, and other qualities, which are not essential to body." § This was certainly a far advance towards the succeeding idealism of some of the Continental schools, if not idealism itself. In regard to mind or soul, "it is my nature," says our philosopher, "that I am a thinking being, which is called mind, soul, intellect, reason; and this nature is more known to me than the nature of my body is. This I clearly and distinctly perceived."|| "I concluded that I was a substance of which the whole essence or nature is only thought."¶ His language in some places certainly identifies mind with thought, like some of the later German speculations. He says (perhaps ambiguously) that "thought ought not otherwise to be conceived of than as thinking substance itself."** But there is a want of uniformity and consistency in his definitions both of mind and matter. For while he in some passages as clearly identifies matter with extension, and mind with thought, as language can do it, at other times he plainly speaks of extension and thought as properties. He even calls them "modes of substances," nay "properties of substances." It is safer to hold in abeyance the charge of decided idealism against Descartes, since his language on the above subjects thus vacillates; though not a

* Principia, Pars I. † Ibid. II. 8.
‡ Est igitur materia et extensio idem. Ibid. 21.
§ Principia, I., II. || Ibid. I., also, Discours de la Méthode. ¶ Ibid. IV.
** Cogitatio et extensio non aliter concipi debent quam ipsa substantia cogitans, et substantia extensa. Princip. I. 63.

few of his statements with regard to substances, extension, and thought, might evidently excuse such a charge.

Our author states, in his Sixth Meditation, that in speculating on the existence of a material world he found that he could, without much difficulty, suppose himself to be deceived in his belief in sensible objects around him. Even their independence on his will did not seem decisive of their existence ; for was it not possible that, in himself, there might be a faculty, though unknown to his consciousness, producing those phenomena ? Every student of the German philosophy must at once perceive the identity of this hypothetical statement with the avowed theory of Fichte. Our author, however, finally decided on admitting an outward universe on the ground of the Divine veracity. "As God has given me a very strong inclination to believe that these ideas (of material things) arise from corporeal objects, I do not see how he could be vindicated from the charge of deceit, if in truth they proceeded from any other source, or were produced by other causes than corporeal things; accordingly it must be concluded that corporeal objects exist." Now we confess to having no *penchant* towards the pure idealism of Berkeley, or the pseudo-idealism of Malebranche;* much less are we enamoured of the Fichtean pantheistic egoism, with its self-created phantasmagoria: yet we can hardly think that the question about the nature of external agencies, whether they are material or only dynamical, or nothing less than the immediate actings of the Creator himself, can fairly be said to have anything to do with his veracity, be the question determined as it may; for either of those speculations may very well consist with morality and religion, whatever may be said of the ingenious idealistic romance of Fichte. We fear that Descartes' argument respecting our own clear and distinct conceptions, in connexion with the veracity of the Deity, would prove rather too much, if we may judge from the history of human opinions.† In regard to the communication which takes place between the soul and the body, Descartes supposed a very subtile fluid,

* Malebranche admitted in theory the real existence of external objects; but his principle, *nous voyons tout en Dieu,* practically discarded matter by excluding it from our perceptions.

† Of course we have no doubt of the veracity of the human faculties in their proper sphere; we have, indeed, nothing else to trust to for our knowledge. The only question here is, how far can they penetrate into the mysteries of nature, of which, undoubtedly, the ultimate constitution of the outward universe is one? There is a hackneyed argument against Descartes' proof, of another kind: it is said he proves a Deity from the veracity of our faculties, and then proves the veracity of our faculties from a Deity. But how could he or any one else prove a Deity but by arguments based on the reliableness of certain psychological principles—i. e., of the human faculties? and must not every theist believe that God is true, and no deceiver? The only question is—could the Deity be implicated in any errors into which his creature might fall, in an attempt to get behind the scenes of the creation? We think not.

the *spiritus animales*, secreted from the blood, and circulating in the "tubular nerves:" in this way external objects affected the soul, resident in the pineal gland of the brain; and the commands of the soul were conveyed, in the contrary direction, to the muscles, in voluntary motion. From this hypothesis we still retain the phrase *animal spirits*, though with another meaning.

On the subject of the Divine agency in the universe, our philosopher held that mind and matter only continue to exist by the perpetual aid (*assistentia*), and co-operation (*concursus*) of the Deity, a doctrine which must of course be admitted, in some form or other, by every Theist. Descartes said that the whole creation depends, for its subsistence, as much as for its original existence, on the *vis creatrix*, or productive agency of the Creator. President Edwards expressed the same views still more strongly, when he said that the continued existence of the moon amounted to a perpetual re-creation of that orb. Geulinx, of Antwerp, endeavoured to deduce, from the doctrine of *concursus*, that of *occasional causes*, or the principle that the Deity is the real author of all the movements, both of finite minds and of bodies, and that there is nothing in them but certain occasions on which he acts. We cannot see in the doctrine of Descartes, as above stated, any necessary germ of Pantheism, as some theorists pretend. That the *occasional causes* of De la Forge and Geulinx may have suggested the *Pre-established Harmony*, and *Optimism*, as maintained by Leibnitz, is very possible. Of Descartes, it is fair to say, he is clear in asserting human freedom, though his genius for abstract speculation led him to dwell more on ideas than on action. He justly remarks, that the mind is free in its volitions, because it is conscious of freedom—the strongest of all arguments.

Descartes is, in some respects, the Bacon of the continental schools, much as he deviated from his prototype. He gave the first impulse to their speculative psychology. The philosophic rationalism of the Cartesian metaphysics, in its *à priori* method, is still pointed to by the Germans as that to which the spirit of their leading systems may ultimately be traced back. He was the first among the moderns to apply the Baconian idea of *observation* distinctly to the operations of thought in consciousness; and in thus isolating thought from all that is not thought, in distinguishing it from all material analogies and adjuncts, Descartes, as Dugald Stewart has remarked, "laid the foundation-stone of the experimental philosophy of the human mind." That the superstructure which he attempted to rear on this basis was not always homogeneous with it, must be admitted by all competent judges. The very idea, however, at his time of day (while the mists of the middle ages were still damp and bewildering on men's intellects) of making a sort of *tabula rasa* of his mind, unlearning all that he had learned, and beginning afresh with the horn-book of knowledge, was a noble aspiration, and worthy of a great genius; and, if the issue did not altogether fulfil the omen of the beginning, we must remember that *to err is human.* His zeal in determining to consult diligently the actual facts of consciousness for himself, merits high praise; notwithstanding his frequent failure in the application of his deductive method, often from his not seeing that it was not applicable to the case; of which some of his physical speculations present flagrant examples. Admirably as he set out, by paying homage to the supremacy of consciousness, and making doubt the pioneer of certainty, he soon went astray by a too great love of theory; his doubtings were exchanged for credulity, and he fell into manifest inconsistencies. For instance, he sometimes repudiates, in terms, all light from final causes, while, at other times, he admits them.* His notions that all we distinctly and clearly conceive is true—that whatever we find in our ideas must necessarily be in the corresponding external things—that every effect must have the same reality as the cause—are evidently liable to give rise to the highest flights of mysticism and idealism, in other hands. In his relation to the German philosophy, indeed, he has not inaptly been termed the "Father of Modern Idealism." Leibnitz said that the chief merit of Descartes lay in recalling the Platonic, or *à priori* method, in withdrawing men from exclusive attention to the senses, and in reviving the doubting spirit of the ancient Academicians.† Some of the more recent Germans have pursued their speculations by Descartes' general method, with developments of which, no doubt, he never could have dreamed. His aphorism—"we desire to deduce effects from causes, not causes from effects," has been extensively patronized in Germany. His notion of substance was converted, in the hands of Spinoza, into a Pantheism, which made the Deity the only real being in the universe; and his ambiguous way of speaking about mind and thought, so as sometimes to identify them, was much like an anticipation of the absolute idealism of Hegel.

The method of Descartes, in its developments, after its basis has been laid, (as that of all methods must, in consciousness) may be termed the *rational method à priori*, as distinguished from that of Bacon—that of experience and deduction. Consciousness was to furnish principles; reason was to deduce results. All science was to be constructed on the twofold basis of intuition and deduction. Irresistible, unassailable truths, were to form the secure foundation; a procedure akin to the mathematical was to rear the goodly superstructure. Away with the doubtful testimonies of sense—away with the delusive colouring of

* Vid. Traité des Passions; art. 175.

† Leibnitz's Letter to Bierling.

imagination, said our philosopher; let us have the intuitive utterances of consciousness, in the pure intellection of the simple, the eternal, the infinite, the absolute. It is the inward apprehension of distinct and clear conceptions, like those of geometry, that must be the corner-stone of all science.* It is assumed that these conceptions, so pregnant with results, can be readily distinguished from the mere impressions of imagination, however vivid, though not a few of Descartes' theories somewhat mar the hopeful prospect, by proving the contrary. The pure conceptions of the mind are to be so clear as to be necessary; the deductions, too, are to be necessarily drawn. The entire method of all science, psychological and physical, is to be assimilated to mathematical demonstration. You are to follow the rational procedure. How do you know that, on the same base, the angle at the centre of a circle is double that at the circumference? Not by admeasurement, not by induction, not in any way by experience—certainly not: you know it by an inevitable deduction from the very definition of a circle in the abstract, a circle which exists neither in nature nor in art, but only in the intellect. Now it was this procedure of geometricians, Descartes tells us, that suggested to him a universal application of it to all our knowledge; and he does not seem to have sufficiently seen the difficulty of applying a method which appropriately belongs to the notions we have of space, to other and very different subjects. Indeed, to find a universal mathematic was his hobby-horse; and on this steed he tried to ride rough shod over all obstacles.† We have seen that, in proving the existence of a Deity, he passes lightly over any evidence furnished by the universe, with all its harmonies and wonders; like Kant, he neglects it with indifference, as not scientific; he rejects the evidence of final causes, and he rests the burden and the fate of the argument on our bare conception of the infinite and the perfect, as a conception involving the necessity of the existence of God, in the same way as the conception of a triangle, as such, necessarily implies all its properties. Instead of arguing the existence of a Deity from the universe, he prefers an Optimism which argues that the universe must be what it is from the existence of the Deity, known by the conscious existence of the *moi* and its *à priori* ideas. He does not insist that there must be a God from the order of the universe, but that there must be a certain order in the universe, because there is a God. Here, we find Descartes and his school down to the modern Germans, opposed to Bacon and his school down to the followers of Locke and Reid. Bacon would say, observe accurately with your senses and your mind,‡

* *Vid.* Regulæ ad Direct. Ingen., III.

† Discours de la Methode, II. Regulæ ad Direct. IV.

‡ Re vel Mente. Nov. Org. Aph. I.

and then draw your inferences: Descartes speaks of "closing his eyes and shutting his ears," in order more intently to listen to the inward voice of reason.* The school of Bacon is jealous of intuitions where it is not shut up to them, and seeks for experience wherever it can be found: the school of Descartes exalts reason and her intuitions often to a giddy height, where all brains must swim and all vision be dizzy; on experience the school relies as little as possible.

Probably no writer, either in ancient or modern times, ever gave a more powerful impulse to philosophical studies than Descartes; and, to these, mathematical studies especially, may be added. Bacon is called the father of experimental science: Descartes has been termed the father of modern philosophy; and, in the German sense of *philosophy*, at least, this is no doubt true; for psychologists and ontologists in that country, have extensively adhered to his method: indeed he stands at the head of the modern names which have made psychology a science. His fame, however, is soundest and greatest as a mathematician. He touched no mathematical subject without displaying his inventive genius, by opening up a new field of investigation. He re-modelled the science of algebra, and applied it to geometry. He left the geometry of the Greeks far behind, by falling upon the idea of expressing the fundamental property of curves by means of equations between their co-ordinates. His claim to originality in his views on the constitution of equations, and the relation between their roots and co-efficients, has been strenuously contested by Dr. Wallis and others: but the new career which he opened up for the mathematical sciences, entitles him to the highest rank in genius for these pursuits.

The failure of Descartes' method is more flagrant in physics than anywhere else. He persuaded himself, however, that he was perfectly consequent to his *à priori* principles, in maintaining that a *vacuum* is impossible—that the same quantity of motion must always be preserved in the universe, because God is immutable; and from the same attribute he also argues the *inertia* of matter. From these and other principles he deduces his philosophical romance of the *vortices*, as accounting for the celestial motions. Newton, however, showed that the periodical times of all bodies that swim in vortices, must be directly as the square of their distances from the centre; whereas it is found by actual observation of the heavens, that the planets, in moving round the sun, obey quite another law, the squares of their periodic times being always as the cubes of their distances. Colin Maclaurin* remarks, not without irony, that from the manner in which Descartes set out, we might

* Meditat. III.

† Account of Sir Isaac Newton's Philosophical Discoveries.

already form some judgment "how hopeful his project was," of explaining the mysteries of nature by deducing effects from their causes. In justice to our illustrious philosopher, however, and in mitigation of his theory of the planetary motions, it should be remembered that, before his time, there had been no physical astronomy; the orbits of the heavenly bodies were not sought in any given law of force, but always in some sort of machinery, respecting the nature of which there had been many hypotheses. Descartes rejected all these, and adopted the notion of ethereal particles revolving round a centre, like the water in a whirlpool.

---

